# When People With Chronic Conditions Turn to Peers on Social Media to Obtain and Share Information: Systematic Review of the Implications for Relationships With Health Care Professionals

**DOI:** 10.2196/41156

**Published:** 2023-04-17

**Authors:** Emilie Mølholm Kjærulff, Tue Helms Andersen, Natasja Kingod, Mette Andersen Nexø

**Affiliations:** 1 Department of Education Copenhagen University Hospital Steno Diabetes Center Copenhagen Herlev Denmark; 2 Section for Health Services Research, Institute of Public Health, University of Copenhagen Copenhagen Denmark

**Keywords:** patient-physician relationship, social media, internet, health information, diabetes, chronic diseases, systematic review, information-seeking behavior, health information, retrieval, sharing

## Abstract

**Background:**

People living with chronic conditions such as diabetes turn to peers on social media to obtain and share information. Although social media use has grown dramatically in the past decade, little is known about its implications for the relationships between people with chronic conditions and health care professionals (HCPs).

**Objective:**

We aimed to systematically review the content and quality of studies examining what the retrieval and sharing of information by people with chronic conditions on social media implies for their relationships with HCPs.

**Methods:**

We conducted a search of studies in MEDLINE (Ovid), Embase (Ovid), PsycINFO (Ovid), and CINAHL (EBSCO). Eligible studies were primary studies; examined social media use; included adults with any type of diabetes, cardiovascular diseases that are closely linked with diabetes, obesity, hypertension, or dyslipidemia; and reported on the implications for people with chronic conditions–HCP relationships when people with chronic conditions access and share information on social media. We used the Mixed Methods Appraisal Tool version 2018 to assess the quality of the studies, and the included studies were narratively synthesized.

**Results:**

Of the 3111 screened studies, 17 (0.55%) were included. Most studies (13/17, 76%) were of low quality. The narrative synthesis identified implications for people with chronic conditions–HCP relationships when people with chronic conditions access and share information on social media, divided into 3 main categories with 7 subcategories. These categories of implications address how the peer interactions of people with chronic conditions on social media can influence their communication with HCPs, how people with chronic conditions discuss advice and medical information from HCPs on social media, and how relationships with HCPs are discussed by people with chronic conditions on social media. The implications are illustrated collectively in a conceptual model.

**Conclusions:**

More evidence is needed to draw conclusions, but the findings indicate that the peer interactions of people with chronic conditions on social media are implicated in the ways in which people with chronic conditions equip themselves for clinical consultations, evaluate the information and advice provided by HCPs, and manage their relationships with HCPs. Future populations with chronic conditions will be raised in a digital world, and social media will likely remain a strategy for obtaining support and information. However, the generally low quality of the studies included in this review points to the relatively immature state of research exploring social media and its implications for people with chronic conditions–HCP relationships. Better study designs and methods for conducting research on social media are needed to generate robust evidence.

## Introduction

### Background

Since the emergence of the internet, people have retrieved and shared health information on the web. This has accelerated with the widespread adoption of social media platforms such as Facebook, Instagram, Twitter, and YouTube, which are collectively estimated to be used by 4.7 billion people as of 2022 [1]. A substantial number of systematic reviews in recent decades have shown that people with chronic conditions turn to peers on social media to exchange information and support [2-9]. Living with chronic conditions such as diabetes, stroke, and heart failure requires complex self-management in the form of administering medicine, diet, and exercise. To this end, peer-to-peer interactions on social media center on adapting medical treatment recommendations to individuals’ daily lives and the demanding emotional and practical aspects of daily living with a chronic condition [6,7].

Although chronic care models in many societies rely primarily on daily self-management by people with chronic conditions [10,11], clinical care remains crucial. Most people with chronic conditions attend regular clinical appointments and receive professional guidance if these health care services are accessible. Reviews addressing a variety of health conditions have provided insights into how information retrieved on the web and social media is discussed in clinical encounters, including what kind of benefits and drawbacks widespread access to such information might entail for relationships between patients and health care professionals (HCPs) [12-15]. The retrieval of health information on the web and social media can potentially empower patients and enhance their collaboration with HCPs if the information is actively discussed [12,14]. However, it can also lead to potential conflicts if HCPs disapprove of accessing health information on the web and social media or perceive the information as a threat to their professional authority [12-14]. Furthermore, patients are often reluctant to bring up such information in clinical consultations, leaving it largely unarticulated [14,15].

These reviews provide important insights, but they also illustrate knowledge gaps requiring further attention. Although Smailhodzic et al [13] specifically investigated social media, other reviews have primarily focused on health information retrieved from websites. In contrast to websites that may be professionally managed and comprise read-only content, social media allows people to create and share content [16]. These functions enable peer interactions, but they also fuel concerns about the credibility of the information and individuals’ ability to evaluate it [17]. In this sense, social media is a distinct type of resource. Moreover, most reviews include people with a wide range of health conditions, including those who are generally healthy. This contrasts with people with conditions such as diabetes who face lifelong self-management and may have recurring needs for information and support from peers. Finally, although 3 reviews conducted quality assessment of the studies, none discussed the results of those assessments.

### Objectives

Our aim was to systematically review the content and quality of studies examining the implications of the retrieval and sharing of information by people with chronic conditions on social media for relationships with HCPs and summarize existing evidence in a conceptual model. Using the population-exposure-outcome framework, we posed the following research question: what implications does the retrieval and sharing of information by people with chronic conditions on social media have for their relationships with HCPs? We were particularly interested in studies including people with diabetes and chronic conditions that are prevalent comorbidities of diabetes. A close link exists between diabetes and cardiovascular diseases such as stroke and heart failure, and these conditions are among the most common noncommunicable diseases in adults worldwide [18,19]. Furthermore, treatments for these conditions rely on extensive daily self-management, which may entail recurring needs for information and support from peers.

## Methods

### Overview

We followed the PRISMA (Preferred Reporting Items for Systematic Reviews and Meta-Analyses) checklist for our systematic review [20]. The literature search followed the PRISMA-S (PRISMA literature search extension) guidelines. [21]. The protocol was registered in the PROSPERO National Institute for Health Research database before the selection of studies (CRD42020205300).

### Search Strategy

We conducted a systematic search of research studies in the following electronic databases: MEDLINE (Ovid), Embase (Ovid), PsycINFO (Ovid), and CINAHL (EBSCO). We deemed these databases appropriate for this study as MEDLINE and Embase are 2 of the largest biomedical databases, PsycINFO covers psychological and social sciences, and CINAHL indexes nursing literature. All 4 databases are searchable via indexed terms, such as the Medical Subject Headings in MEDLINE. An initial search from inception to October 22, 2020, was followed by an updated search in all 4 databases on January 12, 2022. The search strategy combined Medical Subject Heading terms and free-text words for the key concepts of social media, HCP-patient relationships, and chronic diseases related to diabetes mellitus. A limit excluding MEDLINE journals was applied in Embase and CINAHL, and a limit excluding conference abstracts using double negation elimination (not-not search) was applied in Embase and PsycINFO. An information specialist (THA) developed and conducted the search strategy and managed the searches. The search string was developed in Ovid MEDLINE and subsequently translated to the other databases. We evaluated the search strings by comparing them with similar reviews and by looking for known key articles in the searches. All the retrieved studies were organized on EPPI-Reviewer web (EPPI-Centre), a web application for managing the systematic review process [22]. A detailed description of the search strategy is provided in Multimedia Appendix 1.

### Eligibility Criteria

The eligibility criteria were developed based on the research question formulated using the population-exposure-outcome framework. Eligible studies (1) were primary studies published in peer-reviewed journals; (2) examined peer-to-peer interactions on any type of social media or the experience of using social media for retrieving or sharing disease-related information with peers; (3) included adults with diabetes (any type), cardiovascular diseases that are closely linked with diabetes (eg, stroke and heart failure), obesity, hypertension, or dyslipidemia; and (4) reported empirical results on the implications for relationships between people with chronic conditions and HCPs when people with chronic conditions access and share health information on social media. A detailed description of the inclusion and exclusion criteria is available in Multimedia Appendix 2. Furthermore, study records in languages other than English, Danish, Swedish, and Norwegian (languages spoken by the review team) deemed potentially eligible based on the title and abstract were not included in the synthesis but are listed in Multimedia Appendix 2.

After pilot-testing the eligibility criteria by screening a small number of studies, all studies were screened by title and abstract. The full texts of eligible studies were then screened. In total, 2 authors conducted the screening independently (EMK, NK, or MAN) using the EPPI-Reviewer web tool. The studies were reconciled by 2 authors (EMK, NK, or MAN). In case of disagreement, a third author (THA) was available to discuss study eligibility. Full-text reports were retrieved electronically when possible. Those that were not electronically available were retrieved through a research library (Danish Royal Library). All studies were found, and no authors were contacted.

### Quality Assessment

The selected studies used heterogeneous qualitative, quantitative, and mixed methods designs. We used the Mixed Methods Appraisal Tool (MMAT) version 2018 as it was developed for the appraisal of heterogeneous studies [23]. The MMAT includes 2 general screening questions applicable to all study designs and 5 questions based on the study design (Textbox 1). All questions are rated as “yes,” “cannot tell,” and “no.” In keeping with the MMAT guidelines [23], studies were excluded if we rated one or both of the general questions as “cannot tell” or “no.” Thus, studies that lacked a clear research question or used methods that did not allow researchers to address their stated research question were excluded. Studies that passed the general screening questions were included in the knowledge synthesis regardless of the quality ratings based on the 5 study design questions. Regarding the 5 study design questions, no official guidelines exist for judging the threshold for low-quality studies [23]. We rated study quality on a scale from 0% to 100%. The 5 questions each accounted for 20% of the overall score; a question rated as “yes” added 20% to the overall quality score, whereas “cannot tell” or “no” ratings added no percentage. We deemed studies rated ≤40% as low quality, studies rated 60% as medium quality, and studies rated ≥80% as high quality.

**Mixed Methods Appraisal Tool (MMAT) version 2018 quality assessment screening questions. The quality assessment screening questions presented in this textbox are only those relevant to the assessment of the studies included in this systematic review. All screening questions are available in the MMAT version 2018.**

- General quality screening questions (for all study designs)
  Are there clear research questions?
  Do the collected data allow researchers to address the research question?
- Qualitative
  Is the qualitative approach appropriate to answer the research question?
  Are the qualitative data collection methods adequate to address the research question?
  Are the findings adequately derived from the data?
  Is the interpretation of results sufficiently substantiated by data?
  Is there coherence between qualitative data sources, collection, analysis, and interpretation?
- Quantitative nonrandomized
  Are participants representative of the target population?
  Are measurements appropriate regarding both the outcome and intervention (or exposure)?
  Are there complete outcome data?
  Are the confounders accounted for in the design and analysis?
  During the study period, is the intervention administered (or does the exposure occur) as intended?
- Mixed methods
  Is there an adequate rationale for using a mixed methods design to address the research question?
  Are the different components of the study effectively integrated to answer the research question?
  Are the outputs of the integration of qualitative and quantitative components adequately addressed?
  Are divergences and inconsistencies between quantitative and qualitative results adequately addressed?
  Do the different components of the study adhere to the quality criteria of each tradition of the methods involved?

### Data Extraction

In total, 2 authors (EMK and MAN) extracted the study data, which were organized in Microsoft Word (Microsoft Corp) documents. Extracted data included title, authors, publication year, journal, study design, research question, population characteristics (age and gender distribution of study participants and chronic conditions), and procedures for recruiting participants and selecting social media content for examination. In addition, we extracted results related to the implications for people with chronic conditions–HCP relationships when people with chronic conditions obtain and share information on social media and stated the study limitations. We pilot-tested data extraction on 3 studies to evaluate whether the categories accommodated heterogeneous study designs. Data extraction of results related to the implications for people with chronic conditions–HCP relationships when people with chronic conditions access and share information on social media was conducted independently and subsequently reconciled through discussion to ensure the consistency and relevance of the extracted data.

### Knowledge Synthesis

We conducted a narrative synthesis, which allows for identification of patterns across heterogeneous studies to summarize how they address different aspects of the phenomenon of interest [24]. Initially, EMK and MAN read through the extracted results related to the implications for relationships between people with chronic conditions and HCPs when people with chronic conditions access and share information on social media. They then jointly developed codes to describe and categorize the implications identified from the extracted study results. The codes were revised several times to generate categories and subcategories that captured the essence of the identified implications. No theoretical framework guided this process.

## Results

### Study Selection

**Figure 1:**
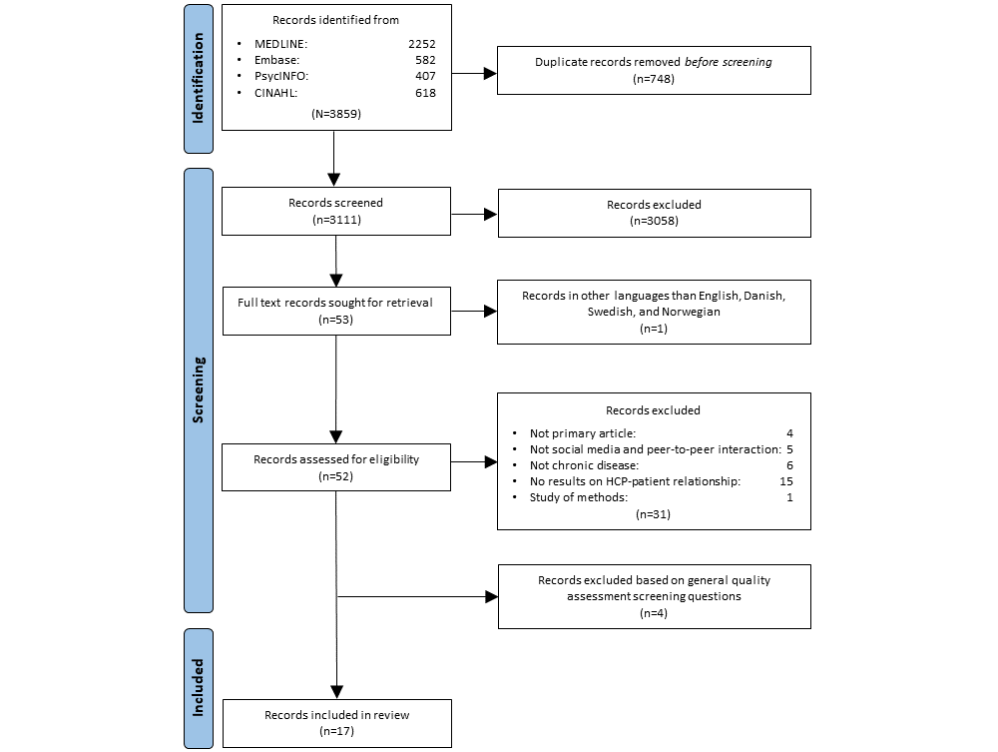
Flowchart showing the selection of studies. HCP: health care professional.

The initial search yielded 3859 studies (Figure 1). The EPPI-Reviewer web tool automatically marked all possible duplicates (742/3859, 19.23%), which were subsequently verified by EMK. References were then manually screened to identify duplicates not detected by the EPPI-Reviewer web tool (6/3859, 0.16%), yielding 3111 unique studies (Figure 1).

On the basis of title and abstract screening, the full texts of 1.7% (53/3111) of the studies were retrieved (Figure 1). Of these 53 studies, we excluded 1 (2%) potentially eligible study (listed in Multimedia Appendix 2) as it was reported in a language other than English, Danish, Swedish, and Norwegian. Thus, the full texts of 52 studies were read to determine eligibility, of which 21 (40%) were included in the quality assessment and 17 (33%) were included in the knowledge synthesis (Figure 1) [25-41].

### Characteristics and Quality of the Included Studies

An overview of the included studies and their quality assessment results is provided in Tables 1 and 2. As stated previously, we deemed studies rated ≤40% as low quality, studies rated 60% as medium quality, and studies rated ≥80% as high quality.

**Table 1:** Characteristics of the included studies (N=17).

Study, year	Design	Population and recruitment	Methods	Findings
Audrain-Pontevia and Menvielle [25], 2018	Cross-sectional web-based survey	328 respondents from OHCs^a^; 39.4% reported having diabetes and other chronic conditions (cancer, asthma, fibromyalgia, Crohn disease, or mental illness) Selection of OHCs not specified. Respondents who declared visiting OHCs on a regular basis throughout the year were selected, but recruitment procedures were not clear	Structural equation modeling and path analysis with 6 hypotheses. Controlled for age, education, employment, and health status Survey items: CMSS^b^ (8 items were adapted from another survey [31]), sense of empowerment (7 items adapted from another survey [31]), patients’ participation during medical consultations (4 items adapted from another survey [42]), and commitment to the relationship with the physician (5 items adapted from another survey [43])	CMSS increased empowerment (PE^c^=0.33; *P*<.001; hypothesis 1 confirmed), participation during consultations (PE=0.27; *P*<.001; hypothesis 2 confirmed), and commitment to the relationship with the physician (PE=0.12; *P*<.05; hypothesis 3 confirmed). Empowerment via CMSS increased participation during consultations (PE=0.45; *P*<.001; hypothesis 4 confirmed), but the commitment to the relationship with the physician decreased (PE=−0.12; *P*<.05; hypothesis 5 not confirmed). Participation during consultations increased commitment to the relationship with the physician (PE=0.40; *P*<.001; hypothesis 6 confirmed).
Audrain-Pontevia et al [26], 2019	Cross-sectional web-based survey	315 respondents from OHCs who reported having diabetes, obesity, and other chronic conditions (HIV, cancer, and other unspecified diseases) Selection of OHCs not specified. Inclusion criteria for respondents were ≥1 chronic disease and having visited OHCs once during the past 3 months, but recruitment procedures were not clear	Structural equation modeling and path analysis with 6 hypotheses Survey items: empowerment (4 items adapted from another survey [42]), patient satisfaction (3 items adapted from another survey [44]), patient commitment (4 items adapted from another survey [43]), and patient compliance (3 items adapted from another survey [45])	Empowerment via social media use increased compliance (coefficient=0.45, *P*<.001; bootstrapping β=.35, *P*<.001; hypothesis 1 confirmed) and patient commitment (coefficient=0.72, *P*<.001; bootstrapping β=.62, *P*<.001; hypothesis 2 confirmed) but not satisfaction (coefficient=−0.01, *P*=.76; bootstrapping β=.05, *P*=.33; hypothesis 3 not confirmed). Satisfaction increased patient commitment (coefficient=0.14, *P*<.001; bootstrapping β=.15, *P*<.01; hypothesis 4 confirmed) but decreased compliance (coefficient=−0.05, *P*<.05; bootstrapping β=−.03, *P*=.39; hypothesis 6 not confirmed). Commitment increased compliance (coefficient=0.54, *P*<.001; bootstrapping β=.51, *P*<.001; hypothesis 5 confirmed).
Bartlett and Coulson [34], 2011	Cross-sectional web-based survey	246 respondents from 33 OSGs^d^ who reported having diabetes and other chronic conditions (eg, arthritis and cancer) Keyword search to identify OSGs; included if focused on chronic conditions. Recruitment procedures for respondents not specified	Multiple regression models and binary regression models. Multiple items averaged to create single variables for each empowerment process and outcome Survey items: demographics (number of items not stated), OSG membership duration and use of the internet for health purposes (number of items not stated), empowerment processes (items adapted from another survey [46] but number of items not reported), empowering outcomes due to OSG participation (items adapted from another survey [46] but number of items not reported), and questions related to relationship with HCPs^e^ and impact of OSG membership on relationship with HCPs (items were inspired by another study [47] but number of items not reported)	Empowerment outcomes (including age, gender, and length of OSG membership) predicted whether OSG users shared information obtained on social media with HCPs (omnibus *χ*^2^_15_=53.0; *P*<.001). A total of 82.2% of respondents had discussed information obtained on the web and social media with their HCP, and 74.2% were either satisfied or extremely satisfied with their HCPs’ reactions. Greater social support exchanged within OSGs (B=−1.107; *P*<.01) or length of membership (B=−0.006; *P*<.01) reduced the likelihood of discussing information obtained on social media with HCPs. Empowerment outcomes predicted whether participants felt that the relationship with their HCP had changed (omnibus *χ*^2^_15_=74.4; *P*<.001). The greater the confidence in their HCP (B=−1.372; *P*<.01), length of membership (B=−0.002; *P*<.05), and age (B=−0.029; *P*<.05), the lower the probability of feeling that their relationship had changed.
Bernhard et al [35], 2017	Qualitative focus group interviews	25 participants with type 2 diabetes Participants recruited from self-help groups, hospitals, and GPs^f^; included if they were German- or Turkish-speaking people with type 2 diabetes and aged ≥18 years	Content analysis [48,49] Theoretical framework: SFMF^g^ [50] Deductive coding via SFMF framework, then inductive coding	Half of the participants had joined social media forums for support and to share information on drug-drug interactions, side effects, new medicines, and tips for adjusting medicine, but participants expressed concerns about the reliability of social media information and their ability to assess the quality of this information. Only a few had discussed information accessed on social media with their physician for clarification.
Bond and Ahmed [36], 2016	Qualitative content analysis	148 threads posted by 127 individuals in 4 social media discussion boards Selection of discussion boards not specified; threads were included if posted within a specific week and relevant to the study purpose	Content analysis [51] No theoretical framework Inductive coding; 10 posts reconciled to ensure validity of coding. Similar codes combined into categories and subcategories	Users exchanged information on how to deal with HCPs, including the right to see the right HCP and how to deal with problems in the patient-HCP relationship. Users also discussed the importance of understanding the information provided by HCPs and the need for questioning HCPs’ advice.
Brookes [37], 2018	Quantitative and qualitative discourse analysis	81 threads consisting of 1072 messages and 119,982 words posted in 3 OSGs for people with type 1 diabetes Keyword search to identify OSGs; threads and messages included if the title or message contained “diabulimia” or “diabulimic”	Corpus-assisted discourse analysis [52] Theoretical framework: medicalization [53] and linguistic patterns resembling medicalizing discourses [54-57] Quantitative analysis [52]: frequency measure to identify words used to linguistically denote diabulimia Qualitative analysis [52]: an examination of the collocates and concordance lines surrounding the words “diabulimia” and “diabulimic” to reveal discourses through which experiences and understandings of diabulimia were construed	Users adopted a position of being an “expert patient” and collectively established diagnostic criteria for diabulimia despite its unofficial disease status (ie, users diagnosed others as having diabulimia or contested the suggestion that users had diabulimia as particular experiences or circumstances failed to satisfy the diagnostic criteria established in the OSG). Users described their experiences of clinical consultations and encouraged others to consult their HCPs to obtain help.
Gilbert et al [38], 2012	Web-based focus groups and web-based cross-sectional survey	42 participants with type 1 diabetes recruited for web-based focus groups; 81 HCPs (diabetes nurse educators, nurses, and others) responding to a web-based survey The Reality Check Community (Australian type 1 diabetes online community) was selected; participants with diabetes were recruited through this community HCP respondents recruited via email through a survey link	Thematic analysis [58] Theoretical framework: Peers for Progress [59] Deductive coding via Peers for Progress framework to create categories and subcategories	HCPs and people with diabetes valued the online community and identified many benefits related to self-management and support. However, people with diabetes reported that HCPs were criticized on social media, which was a negative experience. HCPs reported concerns about inappropriate advice being shared on social media, that diabetes management techniques were not consistent with standard recommendations, and that gossip about HCPs was being shared.
Hewitt-Taylor and Bond [39], 2012	Qualitative content analysis	1583 posts in 126 threads by 561 unique contributors to 4 social media discussion boards for people with diabetes Discussion boards selected based on convenience and activity among members; posts within a specific month were included	Content analysis (no reference stated) No theoretical framework Inductive coding; comparison of codes. Similar codes were merged into categories and subcategories	People with diabetes stressed the importance of a good relationship with HCPs. They valued equal partnerships with HCPs characterized by mutual respect and learning. People with diabetes shared advice on managing relationships with HCPs and on preparing for consultations. They stressed the importance of anticipating HCPs’ responses before the consultation and being clear, confident, and assertive during the consultation but avoiding arguments with HCPs. Some did not share all treatment decisions with HCPs and did not perceive HCPs’ advice as the most reliable or helpful. People with diabetes discussed how HCPs hold a powerful position as gatekeepers, but some expressed a sense of having the power to choose which HCPs they would consult with.
Izuka et al [40], 2017	Qualitative thematic analysis	69 posts on 43 threads by 33 stroke survivors and 17 caregivers The TalkStroke Forum (hosted by the UK Stroke Association) was selected; posts were screened using keywords to select those relevant to the study purpose	Thematic analysis [60,61] No theoretical framework Inductive coding; codes revised into themes. Threads were checked for false information regarding secondary prevention medication [62]	Some patients described strictly following GPs’ advice on medication. Patients described consulting GPs to negotiate new treatments to avoid side effects, and some directed others to consult their GPs to clarify whether side effects were caused by prescribed treatment. Patients described their reactions to GPs’ advice on secondary prevention medicine. Some felt reassured, others remained anxious and described checking for errors in GPs’ prescriptions, and some felt humbled by their GPs’ advice.
Jamison et al [41], 2017	Qualitative thematic analysis	222 posts by 162 stroke survivors, 57 caregivers, and 3 others The TalkStroke forum was selected; posts were screened using keywords to select those relevant to the study purpose	Thematic analysis [60] Theoretical framework: PAPA^h^ [63] Initial coding of posts to generate key themes; these themes were then mapped onto the PAPA framework	HCPs were generally described as having an important role in patients’ trust in secondary prevention medicine and, consequently, adherence. Some users stressed that stopping medication should be on HCP advice, and others described negotiating new treatments with HCPs. Some also raised concerns about HCPs prescribing statins for financial rather than medical reasons.
Johansen et al [27], 2020	Qualitative thematic analysis	16 social media blogs by people with type 1 diabetes Keyword search to identify blogs; included if publicly accessible, in Danish or English, and authorized by individuals aged 18 to 30 years. Selection of blog content not specified	Thematic analysis [60] No theoretical framework 5-step deductive data analysis to identify conceptual patterns and units of meaning relevant to the research question and investigate relationships between codes and levels of themes to finally refine these into overarching themes	Bloggers described the value of HCP support and provided descriptions of support and how they wished to be treated during clinical consultations. They valued HCPs who individualized their approach and comprehended the multifaceted nature of blood glucose monitoring. Several bloggers wanted HCP support for the physical and mental aspects of diabetes. Some found clinical consultations stressful and noted that this stress could be alleviated if HCPs did not judge them by their blood glucose numbers.
Keeling et al [28], 2015	Qualitative analysis and web-based semistructured interviews	Posts in 2 threads in 4 OHCs for people with diabetes (93 posts), breast cancer (167 posts), prostate cancer (185 posts), and depression (522 posts) 45 participants recruited for web-based semistructured member-check interviews (8 people with diabetes, 30 people with breast cancer, 5 people with prostate cancer, and 2 people with depression) Keyword search to identify forums; forums screened using keywords to identify threads relevant to the study purpose. Participants for the interviews were self-selected	Netnographic analysis [64] and content analysis combined with a simple discourse analysis [65-67] Phenomenological approach (no reference) to conducting web-based member-check interviews	Users valued OHCs as a source of information. Most HCPs did not acknowledge this, but a few HCPs engaged in OHCs. Users expressed that sharing medical advice was wrong but sharing experiences was fine; opinions and experiences conflicting with accepted medical views were not necessarily challenged. Users frequently encouraged others to consult their HCPs. They stressed the importance of being able to argue one’s case and raise concerns and preferences in ways that HCPs acknowledged. Most comments about HCPs were positive, but some users did not view GPs as specialists and expressed that they had to accept this and become specialists themselves. Interviewees reflected on their strong dependence on HCPs to filter health information after diagnosis and acknowledged that they lacked HCPs’ professional knowledge.
Litchman et al [29], 2018	Web-based cross-sectional survey	183 respondents with diabetes (type 1, type 2, and latent autoimmune) Survey link posted on various social media platforms; inclusion criteria for respondents: aged ≥18 years and diagnosed with diabetes (type 1, type 2, or latent autoimmune)	Logistic regression Survey scales and items: demographics (11 items) and health history, including self-reported HbA1c^i^ level (8 items), eHealth use (22 items), reasons to join a DOC^j^ (13 items), DOC intensity (8 items adapted from the Facebook intensity scale [68]), DOC engagement (5 items), the internet social capital scale [69] (number of items not stated), health-related quality of life measured using the SF-12v2^k^ [70,71] (12 items), diabetes self-care behaviors measured using SCI-R^l^ [72] (15 items), and source creditability scale [73] (number of items not stated)	Use of DOCs was more intense in respondents who had told their HCPs about their DOC use and felt supported (mean 3.2, SD 0.64) or were unsure if they had felt supported (mean 3.2, SD 0.57) than in those who had never told their HCP about their DOC use (mean 2.6, SD 0.71; *P*<.001). Respondents’ engagement in DOCs was higher for those who had told their HCPs about their DOC use and had felt supported (mean 3.6, SD 1.4) or were unsure if they had felt supported (mean 2.9, SD 1.3) than for those who had never told HCPs about their DOC use (mean 1.9, SD 1.6; *P*<.001). A total of 67.2% of the participants had not informed HCPs about their DOC use.
Litchman et al [30], 2018	Semistructured telephone interviews	20 interviewees with diabetes (type 1, type 2, and latent autoimmune)	Content analysis [74] Theoretical framework for interview guide (not data analysis): theory of intermediation, disintermediation, and apomediation for assessing information credibility [75,76] Codes developed based on 3 transcripts; codes were then applied to all transcripts	DOCs were used for emotional and practical support, enhancing diabetes knowledge, and asking questions deemed too unimportant for HCPs. Participants cross-referenced DOC information with professional sources (eg, Mayo Clinic and professional journals) and consulted their HCPs. DOC use did not replace seeing HCPs. Interviewees consulted HCPs for medical advice and information and stressed that HCPs, not the DOC, should be contacted in case of acute medical issues. However, participants also viewed HCPs as unable to relate to living with diabetes compared with other DOC users and as lacking time and up-to-date information. A few participants only used HCPs for medication prescriptions.
Oh and Lee [31], 2012	Cross-sectional web-based survey	464 respondents from 5 DOCs	Structural equation modeling with covariance matrix tested 4 hypotheses; Sobel test for mediation analysis (hypothesis 4). Controlled for gender, age, education, marital status, employment status, and severity and duration of disease. Confirmatory factor analysis examined internal consistency Survey scales and items: questions related to online community activity (4 items), CMSS (19 items adapted from another study [77]), sense of empowerment measured through 3 subconstructs using PHES^m^ [78,79] and Diabetes Empowerment Scale [80] to modify the PHES (16 items; 6 were used for measuring motivation to achieve disease-related goals, 5 were used for measuring sense of confidence, and 5 were used for measuring sense of control), and intention to actively communicate with the physician (10 items adapted from other studies [81,82])	Online community activity predicted CMSS (β=.27; *P*<.01; hypothesis 1 confirmed). CMSS predicted empowerment (β=.60; *P*<.01; hypothesis 2 confirmed). Empowerment predicted intention to actively communicate with the physician (β=.62; *P*<.01; hypothesis 3 confirmed). The relationship between perceived CMSS and the intention to actively communicate with the physician was mediated by sense of empowerment (z=8.19; *P*<.01; hypothesis 4 confirmed). Sense of empowerment was a valid underlying mechanism to explain how perceived CMSS influences patient intention to actively communicate with the physician. Sociodemographic variables showed partially significant effects on the major variables of the model (*P*<.05).
Willmer and Salzmann-Erikson [32], 2018	Cross-sectional observational study using qualitative content analysis	498 posts from 1 social media forum discussion thread for people with obesity Keyword search to identify forums; forums included if publicly available, if there was active posting (>10 posts daily, >1000 posts in total, and >100 members), and if they were in a Scandinavian language; threads selected if relevant to the study	Qualitative descriptive method [83] No theoretical framework Data coded; codes grouped into categories	Forum members exchanged advice on how to advocate for a referral to specialists from GPs, and forum members described having success after obtaining advice. Forum members described obtaining final approval for the specialist as an exam and prepared themselves by asking for advice from other forum members.
Farnood et al [33], 2021	Qualitative descriptive study and thematic analysis	639 posts from 10 social media forums for people with heart failure Keyword search to identify forums; criteria for including forums not specified; publicly available posts that had been made from 2016 to 2019 were selected	Qualitative descriptive study [83] and thematic analysis [84] Theoretical framework: evidence-based practice guidelines [85] and NPT^n^ [86,87] Data coded and compared; codes grouped into themes and subthemes and mapped onto constructs of NPT	Forum members shared experiences of consultations with HCPs; some were pleased with their HCPs, trusted them, and perceived them as experts. Some described that nurses spoke to them in a relatable manner. Other forum members felt disappointed or frustrated after consultations with HCPs; they had felt rushed, not listened to, that their health issues were not taken seriously, or that the HCP just seemed interested in getting the fee. Consequently, some had lost trust in their HCPs’ knowledge, and some tried to find a new HCP.

^a^OHC: online health community.

^b^CMSS: computer-mediated social support.

^c^PE: parameter estimate.

^d^OSG: online support group.

^e^HCP: health care professional.

^f^GP: general practitioner.

^g^SFMF: Self- and Family Management Framework.

^h^PAPA: Perceptions and Practicalities Approach.

^i^HbA1c: glycated hemoglobin test.

^j^DOC: diabetes online community.

^k^SF-12v2: A shortened form of the SF-36-v2 survey, which is a generic assessment of health-related quality of life from the patient’s perspective.

^l^SCI-R: Self-Care Inventory-Revised.

^m^PHES: psychological health empowerment scale.

^n^NPT: Normalization Process Theory.

**Table 2:** Quality assessment of the included studies (N=17) based on the Mixed Methods Appraisal Tool version 2018.

Study, year	Study design	Q1^a^	Q2^b^	Q3^c^	Q4^d^	Q5^e^	Percentage (0%-100%)^f^
Audrain-Pontevia and Menvielle [25], 2018	Quantitative nonrandomized	C^g^	C	C	Y^h^	Y	40
Audrain-Pontevia et al [26], 2019	Quantitative nonrandomized	C	C	C	C	Y	20
Bartlett and Coulson [34], 2011	Quantitative nonrandomized	C	C	C	N^i^	C	0
Bernhard et al [35], 2017	Qualitative	Y	Y	Y	Y	Y	100
Bond and Ahmed [36], 2016	Qualitative	Y	Y	C	N	N	40
Brookes [37], 2018	Mixed methods	Y	Y	Y	Y	Y	100
Gilbert et al [38], 2012	Mixed methods	C	C	C	N	N	0
Hewitt-Taylor and Bond [39], 2012	Qualitative	Y	C	C	N	N	20
Izuka et al [40], 2017	Qualitative	Y	Y	N	N	N	40
Jamison et al [41], 2017	Qualitative	Y	Y	C	C	C	40
Johansen et al [27], 2020	Qualitative	Y	Y	C	C	C	40
Keeling et al [28], 2015	Qualitative	Y	C	C	C	C	20
Litchman et al [29], 2018	Quantitative nonrandomized	N	C	C	N	N	0
Litchman et al [30], 2018	Qualitative	Y	Y	Y	Y	Y	100
Oh and Lee [31], 2012	Quantitative nonrandomized	C	Y	C	Y	C	40
Willmer and Salzmann-Erikson [32], 2018	Qualitative	Y	Y	C	Y	C	60
Farnood et al [33], 2021	Qualitative	C	Y	N	C	N	20

^a^Q1: quality assessment screening question 1 based on study design (Textbox 1).

^b^Q2: quality assessment screening question 2 based on study design (Textbox 1).

^c^Q3: quality assessment screening question 3 based on study design (Textbox 1).

^d^Q4: quality assessment screening question 4 based on study design (Textbox 1).

^e^Q5: quality assessment screening question 5 based on study design (Textbox 1).

^f^0 to 5 points possible; yes=1, cannot tell=0, no=0; overall quality score shown as a percentage.

^g^C: cannot tell.

^h^Y: yes.

^i^N: no.

### Knowledge Synthesis Results

#### Overview

In total, 3 main categories with 7 subcategories emerged from the narrative synthesis. The results of some studies addressed several implications for the relationships between people with chronic conditions and HCPs when people with chronic conditions access and share information on social media. Therefore, some studies were represented in more than one category or subcategory.

#### How the Peer Interactions of People With Chronic Conditions on Social Media Can Influence Their Communication With HCPs

Studies in the first main category overall suggested that the peer interactions of people with chronic conditions on social media equip them to communicate with HCPs and advocate for desired treatments during clinical consultations. People with chronic conditions may also discuss information obtained from social media with HCPs to evaluate its credibility.

##### Social Media Use and Empowerment in Clinical Consultations

Of the 17 studies, 4 (24%) cross-sectional survey studies [25,26,31,34] suggested that interactions with peers on social media can empower people with chronic conditions to actively participate in clinical consultations, which entails asking questions to engage in dialog with HCPs. For example, Oh and Lee [31] argued that a sense of empowerment was a valid underlying mechanism to explain how perceived support gained from interactions with peers on social media influences the intention of people with diabetes to actively communicate with HCPs. Audrain-Pontevia and Menvielle [25] also described an association between empowerment gained from people with chronic conditions interacting with peers on social media and their participation in clinical consultations. Furthermore, Audrain-Pontevia et al [26] described an association between people with chronic conditions’ empowerment via social media and people with chronic conditions’ commitment to their relationship with HCPs and adherence to HCP treatment recommendations.

We rated these cross-sectional studies as low quality; none defined the target population, making it impossible to assess the representativeness of the samples [25,26,31,34]. Furthermore, some did not provide clear descriptions of their survey items, describe whether the outcome data were complete [25,26,34], or adjust for confounders in their analyses [26,34]. Finally, some of the studies did not provide any descriptions of how they determined whether survey respondents had been “exposed” to peer interactions on social media as intended [31,34] (Table 2).

##### Accessing Information on Social Media to Prepare for Clinical Consultations

Of the 17 studies, 4 (24%) qualitative studies [28,30,32,35] demonstrated that people with chronic conditions accessed information on social media to equip themselves to advocate for specific treatments and medical services in clinical consultations. For example, people with obesity consulted peers on social media to obtain advice on how to request gastric bypass surgery and obtain appointments with medical specialists [32]. People with chronic conditions found it necessary to be informed and prepared when attending clinical consultations, and this included being able to formulate concerns or preferences in ways that HCPs acknowledge [28].

We rated the study by Keeling et al [28] as low quality as it lacked a clear description of the methodological and analytical steps, making it difficult to assess how the results were derived from the data. The study by Willmer and Salzmann-Erikson [32] was rated as medium quality; the authors described using various methods in the abstract and methodology section, but the described analytical procedures matched the cited reference, and the results were sufficiently substantiated by the data. The remaining studies, by Litchman et al [30] and Bernhard et al [35], were rated as high quality as they provided thorough descriptions of the methodological and analytical steps and reported results that were sufficiently substantiated by data, demonstrating coherence between data collection methods, analysis, and interpretation of results (Table 2).

##### Discussing Social Media Information in Clinical Consultations

Of the 17 studies, 3 (18%) qualitative studies [28,30,35] and 2 (12%) cross-sectional studies [29,34] reported that some people with chronic conditions discussed social media information with HCPs to evaluate its credibility. Some people with chronic conditions also depended on HCPs to filter information sourced on social media [28]. Bartlett and Coulson [34] found that most people with chronic conditions discussed social media information with their HCP; 82.2% of survey respondents with various chronic conditions had discussed their information with HCPs, and 74.2% were satisfied with HCPs’ reactions. However, of the 17 studies, 2 (12%) studies suggested that people with chronic conditions tended not to inform HCPs of their retrieval of information from social media [29,35]. For example, as Litchman et al [29] found, 67.2% of survey participants with diabetes had not informed HCPs of their interactions with peers on social media.

As described earlier, the studies by Keeling et al [28] and Bartlett and Coulson [34] were rated as low quality, and the studies by Litchman et al [30] and Bernhard et al [35] were rated as high quality. The remaining cross-sectional survey study, by Litchman et al [29], was rated as low quality—the authors did not define the target population, lacked a representative sample and complete outcome data, and did not adjust for confounders or provide descriptions of how they determined whether survey respondents had been “exposed” to peer interactions on social media as intended (Table 2).

#### How People With Chronic Conditions Discuss Advice and Medical Information From HCPs on Social Media

Studies in the second main category suggested that people with chronic conditions put the information and advice provided by HCPs into perspective by discussing it with peers on social media. For example, people with chronic conditions appropriate and challenge information and advice from HCPs, but they also encourage peers to consult HCPs and follow HCPs’ advice.

##### Challenging Advice and Medical Information From HCPs

Of the 17 studies, 3 (18%) qualitative studies [28,36,39] and 2 (12%) mixed methods studies [37,38] suggested that people with chronic conditions challenged HCP information and advice on social media. People with chronic conditions stressed that one must gain knowledge of one’s condition, question HCPs’ advice, and accept that HCPs’ knowledge has limitations [28,36,39]. As Hewitt-Taylor and Bond [39] described, people with diabetes may not find HCPs’ advice reliable or adequate and may seek a second opinion on their medical issues on social media. Brookes [37] also found that people with diabetes seemed to establish their own diagnostic criteria for diabulimia, which is not formally recognized as a medical condition. In this sense, people with chronic conditions may claim knowledge of medical issues or adopt an “expert role” [37,39]. Furthermore, of the 17 studies, 3 (18%) found that people with chronic conditions may share information and opinions that are not aligned with treatment recommendations [28,37,38].

In addition to the study by Keeling et al [28], the studies by Bond and Ahmed [36], Gilbert et al [38], and Hewitt-Taylor and Bond [39] were rated as low quality. The rationales for the low-quality ratings of the qualitative studies by Bond and Ahmed [36] and Hewitt-Taylor and Bond [39] were the lack of substantial data to support the results, underdeveloped data analysis, and inadequate data to support the interpretation of the results. The study by Gilbert et al [38] did not provide a rationale for using a mixed methods approach, and the different components of the study did not adhere to the quality criteria of each tradition of the methods involved [38]. The remaining mixed methods study by Brookes [37] was rated as high quality as it provided a thorough description of the methodological and analytical steps related to the quantitative and qualitative linguistic approach and integrated quantitative and qualitative components into the analysis (Table 2).

##### Encouraging Peers on Social Media to Consult Their HCPs

Of the 17 studies, 4 (24%) qualitative studies [28,30,40,41] and 1 (6%) mixed methods study [37] found that people with chronic conditions encouraged peers on social media to consult HCPs about issues deemed inappropriate for peers to judge [30,37]. For example, people with chronic conditions stressed that peers on social media should not be consulted about potentially dangerous symptoms [40] or in case of acute illness [30,37]. To help peers clarify questions, people with chronic conditions may also link to other web-based resources managed by HCPs [28]. In this way, people with chronic conditions demonstrated trust in the expertise of HCPs [28,30,41], and some explicitly stated that they followed HCP advice and treatment recommendations and encouraged peers to do the same [40,41].

As discussed previously, the study by Keeling et al [28] was rated as low quality, whereas the studies by Litchman et al [30] and Brookes [37] were rated as high quality. The studies by Izuka et al [40] and Jamison et al [41] were rated as low quality; the authors did not carry out thematic analyses according to the cited references or did not develop analytical themes. Thus, the studies lacked coherence between data collection methods, analysis, and interpretation of results (Table 2).

#### How Relationships With HCPs Are Discussed by People With Chronic Conditions on Social Media

Studies in the third main category suggested that people with chronic conditions use social media to discuss how they experience and manage their relationships with HCPs. Discussions revolved around both a perceived asymmetrical power relationship with HCPs and the value of having a good relationship with HCPs.

##### Power Asymmetry in Relationships With HCPs

Of the 17 studies, 4 (24%) qualitative studies [28,33,39,40] addressed how people with chronic conditions discussed the power asymmetry characterizing their relationships with HCPs. For example, some people with chronic conditions expressed feeling humiliated, subordinated, rushed through clinical consultations, not listened to, and not taken seriously by HCPs [33,39,40]. Consequently, some people with chronic conditions expressed losing trust in HCPs [33] and encouraged peers to switch HCPs if they did not trust their current one [39]. Others questioned whether HCPs prescribed certain drugs for financial reasons rather than medical reasons [33,40]. In this sense, people with chronic conditions discussed the powerful position of HCPs as experts and gatekeepers of health services [28,39,40]. People with chronic conditions also discussed the view that most HCPs do not acknowledge the value of their interactions with peers on social media [28]. However, the only study that included the perspectives of HCPs suggested that they perceive the peer interactions of people with chronic conditions on social media as valuable but are concerned about inappropriate advice or gossip about HCPs shared on social media [38].

As discussed previously, the quality assessment scores of the studies by Keeling et al [28], Hewitt-Taylor and Bond [39], and Izuka et al [40] were low. The study by Farnood et al [33] was also rated as low quality; the authors stated 2 different references for their analysis, introducing methodological conflicts. Furthermore, they did not adequately describe the theoretical framework that was used for the thematic analysis. Therefore, the study lacked coherence between data collection methods, analysis, and interpretation of results (Table 2).

##### The Value of Good Relationships With HCPs

Of the 17 studies, 2 (12%) qualitative studies [27,39] found that people with diabetes stressed the importance of a good relationship with HCPs and shared advice on how to maintain one. People with diabetes described situations in which they felt they received support from HCPs and discussed what kind of relationship they wanted to establish with HCPs and how they expected to be treated by them [27,39]. For example, they stressed the importance of being seen as individuals without judgment from HCPs [27].

As described previously, the study by Hewitt-Taylor and Bond [39] was rated as low quality. The remaining study, by Johansen et al [27], was also rated as low quality. The study cited a reference for thematic analysis but did not provide a theoretical framework for explaining analytical concepts that were used for the deductive thematic analysis. Furthermore, the study lacked a description of how the content from social media blogs was selected, making it difficult to assess the coherence between data collection methods, analysis, and interpretation of results (Table 2).

#### Summary and Conceptual Model

**Figure 2:**
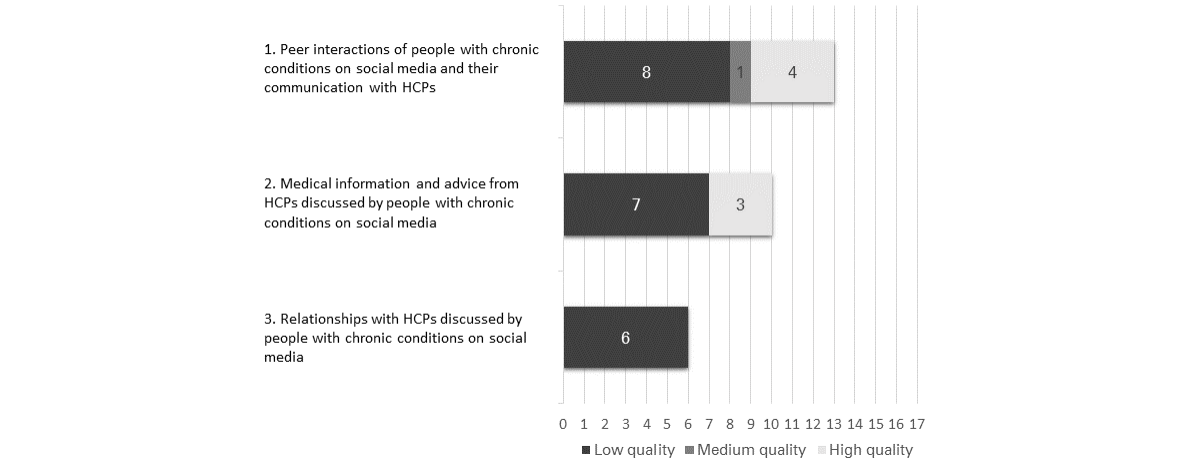
Quality and number of studies supporting the 3 main categories. HCP: health care professional.

The narrative synthesis was based on all study results despite the generally low quality of the included studies. Of the 17 studies, we rated 13 (76%) as low quality, 1 (6%) as medium quality, and 3 (18%) as high quality. An overview of the quality and number of studies supporting the 3 main categories is shown in Figure 2. Studies that were represented in more than one main category or subcategory are also represented more than once in the chart.

**Figure 3:**
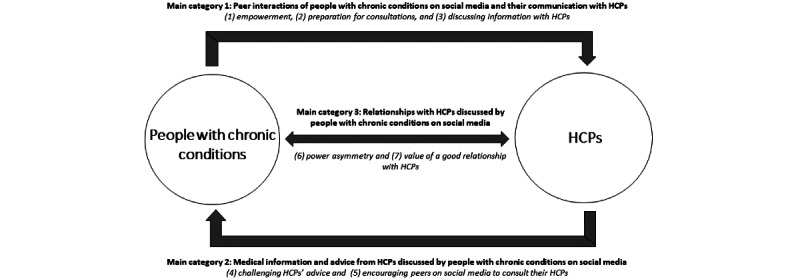
Conceptual model illustrating the implications for people with chronic conditions–health care professional (HCP) relationships when people with chronic conditions access and share information on social media.

To summarize the narrative synthesis, we constructed a conceptual model illustrating the categories and subcategories to demonstrate how they represent implications for people with chronic conditions–HCP relationships when people with chronic conditions access and share information on social media (Figure 3). The model indicates how the peer interactions of people with chronic conditions on social media are implicated in the ways in which people with chronic conditions equip themselves for clinical consultations, evaluate the information provided by HCPs, and manage their relationships with HCPs. Furthermore, the model indicates a flow of information between social media and clinical consultations prompted by people with chronic conditions.

## Discussion

### Principal Findings

To the best of our knowledge, ours is the first study to systematically review the content and quality of studies reporting the implications for people with chronic conditions–HCP relationships when people with chronic conditions access and share information on social media. We identified implications in 3 main categories with 7 subcategories, collectively illustrating them in a conceptual model (Figure 3). Each of the 3 main categories of implications was supported by a different number of studies with varying quality ratings (Figure 2).

The generally low quality of the included studies points to the immature state of research exploring social media. Most notably, the included quantitative studies were all rated as low quality. These studies used cross-sectional survey designs. Generally, the selection of social media communities and recruitment procedures of respondents were unclear, making it difficult to assess whether the samples were representative of the target population. Therefore, the reliability and validity of the included quantitative studies are questionable, and their findings cannot be generalized. Qualitative studies rated as low quality generally lacked clear descriptions of the methodological and analytical steps. In addition, some did not follow the cited research practices. For example, some authors stated that data were coded inductively in accordance with the thematic analysis by Braun and Clarke [60], but they did not develop data-driven, analytical themes. Other authors developed analytical themes but did not describe the analytical concepts or theoretical framework, leaving the analysis underdeveloped. Finally, the mixed methods studies also had varying quality ratings. Altogether, too few studies had sufficient theoretical and methodological transparency, calling into question the validity of the results.

This calls for better research designs and methods for investigating peer-to-peer interactions on social media. For example, quantitative studies should aim for a better sampling strategy and a longitudinal design with control groups that can generate more robust evidence. Qualitative studies should apply methods with greater rigor and transparency and use analytical concepts to build theory. Mixed methods studies could use designs such as convergent, explanatory, or exploratory sequential designs.

### Comparison With Other Reviews

Despite the generally low quality of the studies, many of our findings are reflected in other reviews. For example, Smailhodzic et al [13] concluded that social media use empowers patients and stimulates more equal communication with HCPs. The first main category of implications in this review also points to ways in which the peer interactions of people with chronic conditions on social media can influence communication with HCPs. Studies within subcategory 1 shed light on how peer-to-peer interactions on social media may empower people with chronic conditions in their communication with HCPs, but we are unable to draw conclusions about this association because of study quality. Studies of mixed quality within subcategory 2 also shed light on how peer interactions on social media may equip people with chronic conditions to communicate with HCPs and advocate for desired treatments in clinical consultations.

Furthermore, people with chronic conditions may discuss social media information with HCPs to evaluate its credibility, as suggested in the studies of mixed quality within subcategory 3. Other reviews have concluded that the retrieval of information from web-based resources such as social media can enhance collaboration and relationships between people with chronic conditions and HCPs if the information is discussed in clinical encounters [13,14]. However, judging from other reviews and studies, people with chronic conditions may be reluctant to present their information because of the potentially negative reactions from HCPs [12,13,15,88,89]. Therefore, HCPs may not realize that people with chronic conditions are equipped with information from peers on social media unless they actively address this subject in clinical consultations.

Studies included in the second main category overall suggested that medical information and advice provided by HCPs are discussed and put into perspective on social media. Although most studies included in subcategories 4 and 5 were rated as low quality, the findings highlight how social media allows people with chronic conditions to access and evaluate medical information outside clinical settings. People with chronic conditions may challenge HCPs’ advice, but they may also encourage peers to consult HCPs about questions that are deemed inappropriate for peers to answer. As other reviews and studies have addressed, the user-generated and endless landscape of health information on social media entails complex questions regarding the credibility of social media information [17], but it also facilitates easy access to information adapted to meet individual needs [6,7,90]. Understanding how these dynamics of social media influence relationships with HCPs requires further research.

Finally, studies included in the third main category hint at an implication that other reviews have not addressed. The studies suggested that people with chronic conditions use social media to discuss how they experience and manage their relationship with HCPs. These peer-to-peer interactions revolve around both the perceived asymmetrical power relationship with HCPs and the value of a good relationship with HCPs. On the one hand, these findings indicate that people with chronic conditions may use social media as an outlet for frustration, challenges, and perceived injustice associated with their dependence on HCPs. This could potentially have a negative influence on people with chronic conditions’ trust in HCPs. In contrast, peer-to-peer interactions on social media may also help people with chronic conditions reflect on their experiences of consulting HCPs and support them in establishing a collaborative relationship with HCPs. Given that all studies within subcategories 6 and 7 were rated as low quality, there is a need for more research to understand how such discussions among peers on social media can influence relationships with HCPs.

The identified implications for people with chronic conditions–HCP relationships when people with chronic conditions access and share information on social media are particularly important considering the growing use of social media worldwide. Studies have shown that social media has an impact on people’s health behaviors and decisions, not least in the wake of the COVID-19 pandemic [9,91]. There is also growing evidence that social media use among people with chronic conditions can lead to better clinical and psychosocial outcomes, as described in several reviews [8,92-94]. Although some of these reviews call for more studies, their findings are promising. The internet and social media will likely remain an essential strategy for retrieving health information. In addition, future generations of people with chronic conditions will be raised in a digital world, making it reasonable to believe that social media will be a part of their strategies for handling daily self-management. This emphasizes the need for research that helps elucidate what the retrieval and sharing of health information on social media implies for the relationships between people with chronic conditions and HCPs.

### Strengths and Limitations

A primary strength of this review is the comprehensiveness of our search inquiry. However, there are inherent limitations associated with the inconsistent terminology applied in studies that addressed our research question. Therefore, we performed free-text searches encompassing key concepts, including various terms for describing social media, HCP-patient relationships, and chronic diseases related to diabetes.

We narrowed our scope to focus on diabetes and chronic conditions that are prevalent comorbidities of diabetes. We did not exclude studies that also included people with other chronic diseases, but this scope may have omitted potentially high-quality studies that focused exclusively on chronic conditions other than those of our particular interest.

The narrative synthesis allowed us to identify patterns across studies with heterogeneous designs [24]. To do so in a systematic and transparent way, we grouped studies based on their implications for people with chronic conditions–HCP relationships when people with chronic conditions access and share information on social media. However, the extent to which the included studies addressed our research question in a relevant way varied, reflected in the fact that some studies are represented in more than one category or subcategory of implications.

Consistent with the MMAT guidelines, we excluded studies based on the 2 general screening questions during the quality assessment [23]. Although the MMAT is appropriate for assessing the quality of heterogeneous studies, it posed certain challenges in terms of study selection. For example, studies could pass the general screening questions but be of low quality, whereas other studies could be of reasonable quality but not pass the general questions as they did not pose a clear research question. As most of the included studies (13/17, 76%) were of low quality, the generalizability of our synthesis is limited. However, as noted in the Introduction section, no reviews thus far have discussed the quality of studies addressing our research question, making our review an important contribution.

### Conclusions

Implications for people with chronic conditions–HCP relationships when people with chronic conditions access and share information on social media can be divided into three main categories with 7 subcategories addressing (1) how the peer interactions of people with chronic conditions on social media can influence their communication with HCPs, (2) how people with chronic conditions discuss advice and medical information from HCPs on social media, and (3) how relationships with HCPs are discussed by people with chronic conditions on social media. The findings of this review are particularly important in light of the growing use of social media worldwide. Future populations of people with chronic conditions will be raised in a digital world, making it reasonable to believe that social media will remain a strategy for self-management of chronic conditions. However, the generally low quality of the studies included in this review points to the underdeveloped state of research exploring social media and its implications for people with chronic conditions–HCP relationships. Better study designs and methods for conducting research on social media are needed to generate robust evidence. For example, quantitative studies should aim for a better sampling strategy and a longitudinal design with a control group. Qualitative studies should apply methods with greater rigor and transparency and use analytical concepts to build theory.

## Appendix

Multimedia Appendix 1 Search documentation.

Multimedia Appendix 2 Inclusion and exclusion criteria.

## Abbreviations

HCP: health care professional
MMAT: Mixed Methods Appraisal Tool
PRISMA: Preferred Reporting Items for Systematic Reviews and Meta-Analyses
PRISMA-S: Preferred Reporting Items for Systematic Reviews and Meta-Analyses literature search extension
