# Supplementary material for: When People With Chronic Conditions Turn to Peers on Social Media to Obtain and Share Information: Systematic Review of the Implications for Relationships With Health Care Professionals
Source: J Med Internet Res. 2023 Apr 17;25:e41156. doi: 10.2196/41156 (PMC10152331; doi:10.2196/41156)
Supplement: Multimedia Appendix 1 [file jmir_v25i1e41156_app1.docx]

Search documentation

Key concepts in the search strategy

Concept 1: Social Media

The concept ‘Social media’ was searched for in two parts:

1. Relevant subject headings, e.g. the Medical Subject Heading (MeSH) *Social Media.*
2. Free-text search in the title, abstract and keyword fields. We searched for established concepts e.g. *social media* and *chat*, key terms e.g. *online and* network combined with a proximity operator and well-known social networks, e.g. *Facebook and Twitter*.

Concept 1: Professional-patient relationship

The concept ‘Professional-patient relationship’ was meant to capture references where the relationship between professionals and patients were being studied. Because the word *relationship* and its synonyms were not used consistently in relevant references, we had to split this search block into two new blocks which were then compiled:

1. Subject headings directly relevant to the relationship between professionals and patients, e.g. the Medical Subject Heading (MeSH) *Professional-Patient Relations.*
2. A search aiming to find references including both healthcare professionals and patients. This search contained:
   1. A search on healthcare professionals consisting of:
      1. Relevant subject headings, e.g. the MeSH term *Health Personnel*.
      2. Free-text search in the title, abstract and keyword fields. We searched for key terms, e.g. *care* and *personnel* combined by a proximity operator, Adj3. We also searched for key professions, e.g. *endocrinologist*. All was truncated to include different word endings.
   2. A search on patients consisting of:
      1. Relevant subject headings, e.g. the MeSH term *Patients*.
      2. Free-text search in the title, abstract and keyword fields. We searched for key terms, e.g. *patient* and *client* etc.

Concept 3: Chronic diseases or conditions related to diabetes

The concept ‘Chronic diseases’ was used to capture studies on chronic diseases or conditions related to diabetes. Consequently, we did not search specifically for all chronic diseases but only for *diabetes, cardiovascular disease, dyslipidemia and overweight. All four diseases/conditions were searched for in two parts*:

1. Relevant subject headings, e.g. the Medical Subject Heading (MeSH) *Diabetes.*
2. Free-text search in the title, abstract and keyword fields. We searched for multiple synonyms and acronyms including *diabetes*, *prediabetes*, *T1D*, *T2D* etc. All was truncated to include different word endings.

Information sources and methods

Initially, we searched the databases MEDLINE (Ovid), Embase (Ovid), PsycINFO (Ovid) and CINAHL (EBSCO) from inception to 22 October 2020. An updated search in all four databases was conducted on 12 January 2022.

The MEDLINE search is conducted in the Ovid MEDLINE All <1946-present> database containing the following besides MEDLINE: “**Publisher”, “In-Data-Review”, “In-Process” and “PubMed-not-MEDLINE” from the National Library of Medicine (NLM). The** Ovid MEDLINE All <1946-present> database contains the equivalent content to PubMed.

Searches and results

All complete search strategies with results and comments are listed in table 1. This includes the added date limit used for the updated search.

Table 1 Searches in the databases MEDLINE, Embase, PsycINFO and CINAHL

| # | [MEDLINE (Ovid)] | | # | [Embase (Ovid)] | | # | [PsycINFO (Ovid)] | | # | [CINAHL (EBSCO)] | | Comments |
| --- | --- | --- | --- | --- | --- | --- | --- | --- | --- | --- | --- | --- |
| *Social media* | | | | | | | | | | | | |
| 1 | Social Media/ | | 1 | social media/ | | 1 | exp Social Media/ | | 1 | (MH "Social Media+") | | Subject headings for the phenomenon of interest (social media). |
|  | *8,451* | |  | *22,495* | |  | *15,056* | |  | *15,819* | |  |
| 2 | Online Social Networking/ | | 2 | online social network/ | |  |  | | 2 | (MH "Online Social Networking") | | Subject headings for the phenomenon of interest (online social networking). |
|  | *192* | |  | *334* | |  |  |  |  | *308* | |  |
|  |  |  |  |  |  |  |  |  | 3 | (MH "Instant Messaging") | | Subject headings for the phenomenon of interest (instant messaging). |
|  |  |  |  |  |  |  |  |  |  | *285* | |  |
| 3 | (social adj3 media?).ti,ab,kf,kw. | | 3 | (social adj3 media?).ti,ab,kw. | | 2 | (social adj3 media?).ti,ab. | | 4 | ((TI social OR AB social) N2 (TI media# OR AB media#)) | | Free-text terms for the phenomenon of interest (social media). This search line finds references where *social* and *media* is places at a maximum distance of three words. It has its own line because the word *media* combined with many other words generates numerous references of no relevance. |
|  | *13,707* | |  | *18,831* | |  | *12,919* | |  | *11,339* | |  |
| 4 | ((communit$ or forum$ or group$ or network$ or social$) adj3 (digital or internet or online or software$ or web$)).ti,ab,kf,kw. | | 4 | ((communit$ or forum$ or group$ or network$ or social$) adj3 (digital or internet or online or software$ or web$)).ti,ab,kw. | | 3 | ((communit$ or forum$ or group$ or network$ or social$) adj3 (digital or internet or online or software$ or web$)).ti,ab. | | 5 | (((TI communit* OR AB communit*) OR (TI forum* OR AB forum*) OR (TI group* OR AB group*) OR (TI network* OR AB network*) OR (TI social* OR AB social*)) N2 ((TI digital OR AB digital) OR (TI internet OR AB internet) OR (TI online OR AB online) OR (TI software* OR AB software*) OR (TI web* OR AB web*))) | | Free-text terms for the phenomenon of interest (social media). This search line combines synonyms for groups and synonyms for digital with a proximity operator. |
|  | *16,319* | |  | *21,822* | |  | *16,068* | |  | *9,834* | |  |
| 5 | (blog$ or chat$ or "health 2.0" or image shar$ or instant messag$ or "medicine 2.0" or microblog$ or social networking site$ or social networking website$ or social networking service$ or sns or vlog$ or video sharing or "web 2.0").ti,ab,kf,kw. | | 5 | (blog$ or chat$ or "health 2.0" or image shar$ or instant messag$ or "medicine 2.0" or microblog$ or social networking site$ or social networking website$ or social networking service$ or sns or vlog$ or video sharing or "web 2.0").ti,ab,kw. | | 4 | (blog$ or chat$ or "health 2.0" or image shar$ or instant messag$ or "medicine 2.0" or microblog$ or social networking site$ or social networking website$ or social networking service$ or sns or vlog$ or video sharing or "web 2.0").ti,ab. | | 6 | ((TI blog* OR AB blog*) OR (TI chat* OR AB chat*) OR (TI "health 2.0" OR AB "health 2.0") OR (TI "image shar*" OR AB "image shar*") OR (TI "instant messag*" OR AB "instant messag*") OR (TI "medicine 2.0" OR AB "medicine 2.0") OR (TI microblog* OR AB microblog*) OR (TI "social networking site*" OR AB "social networking site*") OR (TI "social networking website*" OR AB "social networking website*") OR (TI "social networking service*" OR AB "social networking service*") OR (TI sns OR AB sns) OR (TI vlog* OR AB vlog*) OR (TI "video sharing" OR AB "video sharing") OR (TI "web 2.0" OR AB "web 2.0")) | | Free-text terms for the phenomenon of interest (social media). This search line finds established terms for sharing information online. |
|  | *20,106* | |  | *26,980* | |  | *14,051* | |  | *10,028* | |  |
| 6 | (facebook$ or instagram$ or kuaishou$ or linkedin$ or myspace$ or patientslikeme$ or pinterest$ or qzone$ or reddit$ or sinaweibo$ or snapchat$ or tiktok$ or tweet$ or twitter$ or vkontakte$ or whatsapp$ or wechat$ or youtube$).ti,ab,kf,kw. | | 6 | (facebook$ or instagram$ or kuaishou$ or linkedin$ or myspace$ or patientslikeme$ or pinterest$ or qzone$ or reddit$ or sinaweibo$ or snapchat$ or tiktok$ or tweet$ or twitter$ or vkontakte$ or whatsapp$ or wechat$ or youtube$).ti,ab,kw. | | 5 | (facebook$ or instagram$ or kuaishou$ or linkedin$ or myspace$ or patientslikeme$ or pinterest$ or qzone$ or reddit$ or sinaweibo$ or snapchat$ or tiktok$ or tweet$ or twitter$ or vkontakte$ or whatsapp$ or wechat$ or youtube$).ti,ab. | | 7 | ((TI facebook* OR AB facebook*) OR (TI instagram* OR AB instagram*) OR (TI kuaishou* OR AB kuaishou*) OR (TI linkedin* OR AB linkedin*) OR (TI myspace* OR AB myspace*) OR (TI patientslikeme* OR AB patientslikeme*) OR (TI pinterest* OR AB pinterest*) OR (TI qzone* OR AB qzone*) OR (TI reddit* OR AB reddit*) OR (TI sinaweibo* OR AB sinaweibo*) OR (TI snapchat* OR AB snapchat*) OR (TI tiktok* OR AB tiktok*) OR (TI tweet* OR AB tweet*) OR (TI twitter* OR AB twitter*) OR (TI vkontakte* OR AB vkontakte*) OR (TI whatsapp* OR AB whatsapp*) OR (TI wechat* OR AB wechat*) OR (TI youtube* OR AB youtube*)) | | Free-text terms for the phenomenon of interest (social media). This search line entails the biggest social networks at the time of the search. |
|  | *10,860* | |  | *14,792* | |  | *9,465* | |  | *10,314* | |  |
| 7 | or/1-6 | | 7 | or/1-6 | | 6 | or/1-5 | | 8 | S1 OR S2 OR S3 OR S4 OR S5 OR S6 OR S7 | |  |
|  | *52,037* | |  | *73,807* | |  | *42,624* | |  | *39,952* | |  |
| *Professional patient relationship* | | | | | | | | | | | | |
| 8 | exp Professional-Patient Relations/ | | 8 | exp professional-patient relationship/ | |  |  |  | 9 | (MH "Professional-Patient Relations+") | | Subject headings for the population of interest (professional-patient relationship). |
|  | *143,028* | |  | *47,749* | |  |  |  |  | *96,526* | |  |
| 9 | Professional-Family Relations/ | |  |  |  |  |  |  | 10 | (MH "Professional-Family Relations") | | Subject headings for the population of interest (professional-family relationship). |
|  | *14,937* | |  |  |  |  |  |  |  | *16,658* | |  |
| 10 | Hospital-Patient Relations/ | |  |  |  |  |  |  |  |  |  | Subject headings for the population of interest (hospital-patient relationship). |
|  | *1,846* | |  |  |  |  |  |  |  |  |  |  |
|  |  |  |  |  |  |  |  |  | 11 | MH "Professional-Client Relations+") | | Subject headings for the population of interest (professional-client relationship). |
|  |  |  |  |  |  |  |  |  |  | *7,669* | |  |
| 11 | exp Patient Acceptance of Health Care/ | | 9 | patient attitude/ | | 7 | exp client attitudes/ | |  |  |  | Subject headings for the population of interest (patient attitude). |
|  | *154,058* | |  | *68,609* | |  | *22,439* | |  |  |  |  |
| 12 | or/8-11 | | 10 | 8 or 9 | |  |  |  | 12 | S9 OR S10 OR S11 | |  |
|  | *295,782* | |  | *115,037* | |  |  |  |  | *117,070* | |  |
| 13 | exp Health Personnel/ | | 11 | exp health care personnel/ | | 8 | exp health personnel/ | | 13 | (MH "Health Personnel+") | | Subject headings for the population of interest (health personnel). |
|  | *521,450* | |  | *1,589,443* | |  | *163,724* | |  | *584,079* | |  |
| 14 | exp Delivery of Health Care/ | | 12 | exp health care delivery/ | |  |  |  | 14 | (MH "Health Care Delivery+") | | Subject headings for the population of interest (health personnel). |
|  | *1,090,954* | |  | *3,363,266* | |  |  |  |  | *345,762* | |  |
| 15 | exp Health Services/ | | 13 | exp health service/ | | 9 | exp health care services/ | | 15 | (MH "Health Services+") | | Subject headings for the population of interest (health personnel). |
|  | *2,157,972* | |  | *5,547,458* | |  | *211,553* | |  | *1,055,137* | |  |
| 16 | ((care$ or health$ or medical$) adj3 (assistant$ or giver$ or person?el$ or provider$ or worker$)).ti,ab,kf,kw. | | 14 | ((care$ or health$ or medical$) adj3 (assistant$ or giver$ or person?el$ or provider$ or worker$)).ti,ab,kw. | | 10 | ((care$ or health$ or medical$) adj3 (assistant$ or giver$ or person?el$ or provider$ or worker$)).ti,ab. | | 16 | (((TI care* OR AB care*) OR (TI health* OR AB health*) OR (TI medical* OR AB medical*)) N2 ((TI assistant* OR AB assistant*) OR (TI giver* OR AB giver*) OR (TI person#el* OR AB person#el*) OR (TI provider* OR AB provider*) OR (TI worker* OR AB worker*))) | | Free-text terms for the phenomenon of interest (health personnel). This search line combines terms to search for different health personnel with a proximity operator. |
|  | *178,580* | |  | *229,995* | |  | *53,030* | |  | *105,324* | |  |
| 17 | (cardiologist$ or caregiver$ or doctor$ or endocrinologist$ or practitioner$ or gp$ or nurse$ or physician$ or professional$).ti,ab,kf,kw. | | 15 | (cardiologist$ or caregiver$ or doctor$ or endocrinologist$ or practitioner$ or gp$ or nurse$ or physician$ or professional$).ti,ab,kw. | | 11 | (cardiologist$ or caregiver$ or doctor$ or endocrinologist$ or practitioner$ or gp$ or nurse$ or physician$ or professional$).ti,ab. | | 17 | ((TI cardiologist* OR AB cardiologist*) OR (TI caregiver* OR AB caregiver*) OR (TI doctor* OR AB doctor*) OR (TI endocrinologist* OR AB endocrinologist*) OR (TI practitioner* OR AB practitioner*) OR (TI gp* OR AB gp*) OR (TI nurse* OR AB nurse*) OR (TI physician* OR AB physician*) OR (TI professional* OR AB professional*)) | | Free-text terms for the phenomenon of interest (health personnel). This search line finds established health personnel roles. |
|  | *1,309,517* | |  | *1,704,409* | |  | *481,474* | |  | *801,272* | |  |
| 18 | ((care$ or health$) adj3 (delive$ or service$ or system$)).ti,ab,kf,kw. | | 16 | ((care$ or health$) adj3 (delive$ or service$ or system$)).ti,ab,kw. | | 12 | ((care$ or health$) adj3 (delive$ or service$ or system$)).ti,ab. | | 18 | (((TI care* OR AB care*) OR (TI health* OR AB health*)) N2 ((TI delive* OR AB delive*) OR (TI service* OR AB service*) OR (TI system* OR AB system*))) | | Free-text terms for the phenomenon of interest (health personnel). This search line identifies healthcare services. |
|  | *384,272* | |  | *488,923* | |  | *115,615* | |  | *217,799* | |  |
| 19 | or/13-18 | | 17 | or/11-16 | | 13 | or/8-12 | | 19 | S13 OR S14 OR S15 OR S16 OR S17 OR S18 | |  |
|  | *3,933,197* | |  | *7,135,453* | |  | *718,241* | |  | *2,168,907* | |  |
| 20 | exp Patients/ | | 18 | exp patient/ | | 14 | exp patients/ | | 20 | (MH "Patients+") | | Subject headings for the population of interest (patients). |
|  | *66,190* | |  | *2,495,224* | |  | *98,247* | |  | *278,881* | |  |
| 21 | (client$ or consumer$ or diabetic$ or patient$ or people$ or person$ or user$ or famil$).ti,ab,kf,kw. | | 19 | (client$ or consumer$ or diabetic$ or patient$ or people$ or person$ or user$ or famil$).ti,ab,kw. | | 15 | (client$ or consumer$ or diabetic$ or patient$ or people$ or person$ or user$ or famil$).ti,ab. | | 21 | ((TI client* OR AB client*) OR (TI consumer* OR AB consumer*) OR (TI diabetic* OR AB diabetic*) OR (TI patient* OR AB patient*) OR (TI people* OR AB people*) OR (TI person* OR AB person*) OR (TI user* OR AB user*) OR (TI famil* OR AB famil*)) | | Free-text terms for the population of interest (patients). This search line identifies references including synonyms for the word *patient*. |
|  | *8,621,615* | |  | *12,003,493* | |  | *1,870,644* | |  | *2,382,237* | |  |
| 22 | 20 or 21 | | 20 | 18 or 19 | | 16 | 14 or 15 | | 22 | S20 OR S21 | |  |
|  | *8,640,562* | |  | *12,171,533* | |  | *1,882,723* | |  | *2,485,741* | |  |
| 23 | 19 and 22 | | 21 | 17 and 20 | | 17 | 13 and 16 | | 23 | S19 AND S22 | |  |
|  | *1,896,752* | |  | *4,268,466* | |  | *426,543* | |  | *903,764* | |  |
| 24 | 12 or 23 | | 22 | 10 or 21 | | 18 | 7 or 17 | | 24 | S12 OR S23 | |  |
|  | *2,005,308* | |  | *4,323,948* | |  | *437,220* | |  | *961,136* | |  |
| *Chronic disease* | | | | | | | | | | | | |
| 25 | exp Chronic Disease/ | | 23 | exp chronic disease/ | | 19 | exp Chronic Illness/ | | 25 | (MH "Chronic Disease+") | | Subject headings for the population of interest (people with chronic disease). |
|  | *264,716* | |  | *182,245* | |  | *29,629* | |  | *65,661* | |  |
| 26 | Noncommunicable Diseases/ | | 24 | noncommunicable disease/ | |  |  |  |  |  |  | Subject headings for the population of interest (people with Noncommunicable disease). |
|  | *1,338* | |  | *6,357* | |  |  |  |  |  |  |  |
| 27 | (chronic$ or non-communicable$ or noncommunicable$ or non-infectious$ or noninfectious$ or ncd$).ti,ab,kf,kw. | | 25 | (chronic$ or non-communicable$ or noncommunicable$ or non-infectious$ or noninfectious$ or ncd$).ti,ab,kw. | | 20 | (chronic$ or non-communicable$ or noncommunicable$ or non-infectious$ or noninfectious$ or ncd$).ti,ab. | | 26 | ((TI chronic* OR AB chronic*) OR (TI non-communicable* OR AB non-communicable*) OR (TI noncommunicable* OR AB noncommunicable*) OR (TI non-infectious* OR AB non-infectious*) OR (TI noninfectious* OR AB noninfectious*) OR (TI ncd* OR AB ncd*)) | | Free-text terms for the population of interest (people with chronic disease). This search line identifies references including synonyms for *chronic diseases*. |
|  | *1,256,295* | |  | *1,754,978* | |  | *157,932* | |  | *267,298* | |  |
| 28 | exp Diabetes Mellitus/ | | 26 | exp diabetes mellitus/ | | 21 | exp diabetes/ | | 27 | (MH "Diabetes Mellitus+") | | Subject headings for the population of interest (people with diabetes). |
|  | *430,020* | |  | *973,215* | |  | *17,936* | |  | *167,827* | |  |
| 29 | (diabet$ or prediabet$ or t1d$ or t2d$ or lada$ or mody$).ti,ab,kf,kw. | | 27 | (diabet$ or prediabet$ or t1d$ or t2d$ or lada$ or mody$).ti,ab,kw. | | 22 | (diabet$ or prediabet$ or t1d$ or t2d$ or lada$ or mody$).ti,ab. | | 28 | ((TI diabet* OR AB diabet*) OR (TI prediabet* OR AB prediabet*) OR (TI t1d* OR AB t1d*) OR (TI t2d* OR AB t2d*) OR (TI lada* OR AB lada*) OR (TI mody* OR AB mody*)) | | Free-text terms for the population of interest (people with diabetes). This search line identifies references including synonyms for *diabetes*. |
|  | *650,324* | |  | *969,562* | |  | *31,878* | |  | *201,934* | |  |
| 30 | exp Cardiovascular Diseases/ | | 28 | exp cardiovascular disease/ | | 23 | exp cardiovascular disorders/ | | 29 | (MH "Cardiovascular Diseases+") | | Subject headings for the population of interest (people with cardiovascular disease). |
|  | *2,404,411* | |  | *4,119,979* | |  | *62,766* | |  | *602,014* | |  |
| 31 | exp Dyslipidemias/ | | 29 | exp dyslipidemia/ | |  |  |  | 30 | (MH "Hyperlipidemia+") | | Subject headings for the population of interest (people with dyslipidemia). |
|  | *80,222* | |  | *73,081* | |  |  |  |  | *20,895* | |  |
| 32 | (high blood pressure$ or cardiovascular$ or cvd$ or dyslipidemia$ or dyslipidaemia$ or dyslipoproteinemia$ or hypertens$).ti,ab,kf,kw. | | 30 | (high blood pressure$ or cardiovascular$ or cvd$ or dyslipidemia$ or dyslipidaemia$ or dyslipoproteinemia$ or hypertens$).ti,ab,kw. | | 24 | (high blood pressure$ or cardiovascular$ or cvd$ or dyslipidemia$ or dyslipidaemia$ or dyslipoproteinemia$ or hypertens$).ti,ab. | | 31 | ((TI "high blood pressure*" OR AB "high blood pressure*") OR (TI cardiovascular* OR AB cardiovascular*) OR (TI cvd* OR AB cvd*) OR (TI dyslipidemia* OR AB dyslipidemia*) OR (TI dyslipidaemia* OR AB dyslipidaemia*) OR (TI dyslipoproteinemia* OR AB dyslipoproteinemia*) OR (TI hypertens* OR AB hypertens*)) | | Free-text terms for the population of interest (people with cardiovascular disease). This search line identifies references including synonyms for *cardiovascular diseases*. |
|  | *855,553* | |  | *1,252,607* | |  | *44,268* | |  | *203,538* | |  |
| 33 | exp Overweight/ | |  |  |  | 25 | exp overweight/ | |  |  |  | Subject headings for the population of interest (people who are overweight). |
|  | *222,403* | |  |  |  |  | *26,244* | |  |  |  |  |
| 34 | exp Obesity/ | | 31 | exp obesity/ | |  |  |  | 32 | (MH "Obesity+") | | Subject headings for the population of interest (people who are obese). |
|  | *215,931* | |  | *528,716* | |  |  |  |  | *101,078* | |  |
| 35 | (obesit$ or overweight$).ti,ab,kf,kw. | | 32 | (obesit$ or overweight$).ti,ab,kw. | | 26 | (obesit$ or overweight$).ti,ab. | | 33 | ((TI obesit* OR AB obesit*) OR (TI overweight* OR AB overweight*)) | | Free-text terms for the population of interest (people who are overweight). This search line identifies references including synonyms for overweight. |
|  | *285,750* | |  | *429,230* | |  | *39,733* | |  | *95,890* | |  |
| 36 | or/25-35 | | 33 | or/23-32 | | 27 | or/19-26 | | 34 | S25 OR S26 OR S27 OR S28 OR S29 OR S30 OR S31 OR S32 OR S33 | |  |
|  | *4,529,286* | |  | *6,629,742* | |  | *292,856* | |  | *1,157,908* | |  |
| 37 | 7 and 24 and 36 | | 34 | 7 and 22 and 33 | | 28 | 6 and 18 and 27 | | 35 | S8 AND S24 AND S34 | |  |
|  | *1,770* | |  | *4,273* | |  | *431* | |  | *944* | |  |
| *Exclude medline journal limit* | | | | | | | | | | | | |
|  |  |  | 35 | limit 34 to exclude medline journals | |  |  |  | 36 | S8 AND S24 AND S34  (exclude medline journals) | | Limit to exclude MEDLINE journals. |
|  |  |  |  | *429* | |  |  |  |  | *462* | |  |
|  |  |  | 36 | limit 35 to conference abstract | | 29 | limit 28 to peer reviewed journal | |  |  |  | Limits to exclude other than primary research articles. |
|  |  |  |  | *105* | |  | *345* | |  |  |  |  |
|  |  |  | 37 | 35 not 36 | |  |  |  |  |  |  |  |
|  |  |  |  | *324* | |  |  |  |  |  |  |  |
| *Updated search* | | | | | | | | | | | | |
|  | limit 37 to (dt="20201022-20251231" or ez="20201022-20251231") | |  | limit 37 to (dc="20201022-20251231" or rd="20201022-20251231") | |  | limit 29 to up="20201022-20251231" | |  | EM 20201022-20251231 | | Updating search using date filters. |
|  | *482* | |  | *258* | |  | *62* | |  | *156* | |  |
