# Supplementary material for: When People With Chronic Conditions Turn to Peers on Social Media to Obtain and Share Information: Systematic Review of the Implications for Relationships With Health Care Professionals
Source: J Med Internet Res. 2023 Apr 17;25:e41156. doi: 10.2196/41156 (PMC10152331; doi:10.2196/41156)
Supplement: Multimedia Appendix 2 [file jmir_v25i1e41156_app2.docx]

Appendix 2: Inclusion and exclusion criteria

| **Inclusion criteria** | **Exclusion criteria** |
| --- | --- |
| Publication type and study design:  Primary articles published in peer-reviewed journals, written in English, Danish, Swedish, Norwegian language | - Conference abstracts - Studies not subject to peer review - Reviews - Protocols - Studies of methodologies - Animal studies - Studies in other languages than English, Danish, Swedish, and Norwegian are translated through Google Translate (if deemed potentially relevant, studies are included in an appendix, but not included in the synthesis)* |
| Social media: studies must investigate adult peer-to-peer interaction on any kind of social media platform or the experience of engaging in peer-to-peer interactions on any kind of social media platform  Adults are defined as: aged ≥18 years.   Social media platforms are defined as: apps or websites that allow the creation and exchange of user-generated content   Interactions on social media or engaging in peer-to-peer interactions are defined as: communication through text, pictures, videos, symbols (e.g., emojis), reading peers’ interactions on social media  Studies of interventions in which people with chronic conditions must engage in peer-to-peer interactions on social media | - Studies in which ≥50% of the population are not adults - Studies that investigate mainstream media (TV, News Papers) - Apps or websites developed exclusively to deliver telemedicine (e.g., HCP-patient communication services with no peer-to-peer interaction) - Studies investigating the development of apps or websites for patients - Studies in which surveys are disseminated through social media, but social media use is not investigated - Studies in which participants are recruited through social media platforms, but social media use is not studied - Studies on educational material, campaigns, and commercial content delivered through social media platforms or websites |
| Chronic conditions: studies must include people with at least one of the following conditions: diabetes, cardiovascular diseases that are closely linked with diabetes, obesity, hypertension or dyslipidaemia   Diabetes is defined as: diabetes type 1, type 2, LADA, MODY, gestational diabetes  Cardiovascular diseases are defined as: cerebrovascular disease; stroke, transient ischemic attack, coronary artery disease (CAD); myocardial infarction, heart failure, peripheral arterial disease (PAD); diabetic foot, intermittent claudication and microvascular complications (diabetic retinopathy (blindness), diabetic nephropathy, diabetic nephropathy (diabetic kidney disease))  Obesity is defined as: BMI of ≥30  Studies that include several chronic conditions do not have to report results at an individual disease level | - Studies in which ≥50% are caregivers - Studies that do not include people with at least one of the chronic conditions listed in the inclusion criteria |
| Studies must report empirical results on the implications for relationships between people with chronic conditions and health care professionals when people with chronic conditions access and share information on social media  Implications for relationships between people with chronic conditions and health care professionals are defined as (but not limited to): Communication, experiences, perceived benefits or challenges, or health care professionals’ experiences of people with chronic conditions’ social media use for retrieving health information or interacting with peers  Health care professionals are defined as: Nurses, physicians, dieticians, or other professional health care staff providing clinical care for people with chronic conditions | - Studies that only address implications for relationships between people with chronic conditions and health care professionals in their discussions or future study recommendations, but do not report empirical results on implications - Alternative treatments or care services delivered by persons that are not health care professionals - Students of any kind |

*We excluded 1 potentially eligible study as it was reported in a language other than English, Danish, Swedish, and Norwegian:

Pereira Neto, André, Letícia Barbosa, Adriano da Silva, and Monica Lucia Gomes Dantas. 2015. “O Paciente Informado e Os Saberes Médicos: Um Estudo de Etnografia Virtual Em Comunidades de Doentes No Facebook.” *Historia, Ciencias, Saude - Manguinhos* 22: 1653–71. https://doi.org/10.1590/S0104-59702015000500007.
